# Weekly Fluctuations in Internal Load and Neuromuscular Performance Across a 10-Week Training Period in Elite Female Boxers

**DOI:** 10.3390/life16030386

**Published:** 2026-02-28

**Authors:** Ahmet Serhat Aydın, Tolga Altuğ, Coşkun Yılmaz, Adela Badau, Mehmet Söyler

**Affiliations:** 1 PhD Program, Department of Physical Education and Sports, Institute of Health Sciences, Gazi University, 06570 Ankara, Türkiye; serhataydingazi@gmail.com; 2 Department of Sport Sciences Faculty, Ağrı İbrahim Çeçen University, 04100 Ağrı, Türkiye; talgum@agri.edu.tr; 3 Kelkit Aydın Doğan Vocational School, Gümüshane University, 29100 Gümüşhane, Türkiye; coskun.yilmaz@gumushane.edu.tr; 4 Department of Physical Education and Special Motricity, Transilvania University of Brasov, 500068 Brasov, Romania; 5 Vocational School of Social Sciences, Çankırı Karatekin University, 18100 Çankırı, Türkiye

**Keywords:** training load, sRPE, training monotony, neuromuscular performance, female boxers

## Abstract

This study examined weekly internal load and neuromuscular performance in elite junior female boxers over 10 weeks. Internal load was quantified using session rating of perceived exertion (sRPE), from which weekly monotony and strain were derived. Neuromuscular performance was assessed weekly using wall-sit endurance and a repetitive jump test. Twenty elite junior female boxers (Mean ± SD: 18.9 ± 1.2) were monitored during regular training without experimental manipulation. Weekly sRPE-derived training load, monotony, and strain showed statistically significant week-to-week fluctuations (*p* < 0.001). Neuromuscular performance improved in week 2, declined during weeks 3–5, and partially recovered in week 6. The findings demonstrated consistent temporal alignment between internal-load indices and week-to-week neuromuscular performance changes within an observational monitoring framework. Inter-individual variability was observed across athletes. Overall, sRPE-derived indices reflected training stress patterns and were aligned with neuromuscular performance changes in elite female boxers, supporting their use for contextual monitoring of weekly training responses. These findings support the practical integration of internal-load and performance monitoring in elite female combat-sport settings. Future research incorporating boxing-specific external-load metrics, physiological markers, and longer monitoring periods may further refine individualized load-management strategies.

## 1. Introduction

Effective management of training load is critical to enhancing performance while minimizing injury and illness risk in competitive athletes. Recent consensus statements from the International Olympic Committee emphasize that rapid, unplanned increases in training load, insufficient recovery, and poorly structured periodization in relation to competition schedules increase the risk of adverse outcomes, highlighting systematic training-load monitoring as a key component of preventive strategies [1]. In combat sports such as boxing, where repeated high-intensity efforts and congested training microcycles are common, monitoring the balance between training stress and adaptation is particularly important to support both performance sustainability and athlete health.

Among internal load measures, the session rating of perceived exertion (sRPE) has become one of the most widely used tools due to its practicality, low cost, and responsiveness to both acute and chronic training stimuli [2,3]. Indices derived from sRPE, such as weekly training monotony and training strain, summarize the distribution and cumulative impact of training loads and have been proposed as sensitive markers of excessive or poorly managed training stress [3,4]. Recent work across team and individual sports suggests that high strain and elevated monotony may be associated with impaired well-being, increased fatigue, and a higher likelihood of maladaptation when maintained over several weeks [5,6]. At the same time, conceptual critiques of the acute: chronic workload ratio (ACWR) have highlighted important methodological limitations and inconsistent predictive value for injury risk [7,8], reinforcing the need to focus on simple, transparent monitoring metrics with clear interpretability, such as sRPE-derived load, monotony, and strain.

In parallel with internal measures, a wide range of approaches has been developed to quantify external training load and neuromuscular status. In boxing, biomechanical and technology-assisted methods have been used to characterize punch kinematics and force production, often relying on force sensors or wearable accelerometer-based systems [9,10]. While these tools can provide detailed information on striking performance, their cost, technical complexity, and data-processing requirements limit their feasibility for routine, long-term monitoring in many high-performance environments. Consequently, practical monitoring frameworks increasingly combine simple internal-load measures such as sRPE with accessible field-based tests of neuromuscular performance (e.g., jump tests, isometric endurance tasks) that can be repeatedly implemented without major disruption to training [11,12].

At the same time, sex-specific physiological factors are now recognized as important modifiers of the training load–response relationship in female athletes. The menstrual cycle has been associated with perceived changes in fatigue, motivation, and performance in many athletes, although objective performance outcomes show heterogeneous and often equivocal results [13,14]. Low energy availability and Relative Energy Deficiency in Sport (RED-S) have been identified as major threats to both health and performance, with recent IOC consensus statements emphasizing their prevalence and multisystem consequences in female athletes [1]. Together, these findings underline the importance of monitoring training load and performance in women within an integrative framework that acknowledges potential influences of menstrual status, energy availability, and recovery capacity, even when these factors are not directly measured. Accordingly, such internal load indices should be interpreted with caution and not primarily as predictors of performance, fatigue, or injury, but rather as contextual indicators that assist in describing and interpreting training stress fluctuations over time within observational monitoring frameworks [15].

Despite growing attention to female-specific issues, the empirical literature on training load, neuromuscular performance, and adaptation in female boxers remains scarce. Existing studies in boxing have largely focused on biomechanical determinants of punching, strength and power interventions, or short-term performance changes, and often involve mixed-sex cohorts or predominantly male samples [9,16,17].

Although several studies have examined the relationship between training load, fatigue, and performance in boxing, the existing literature remains limited in both scope and population, with a predominant focus on biomechanical outcomes, male athletes, or short-term performance responses [9,18]. To date, no studies have systematically tracked internal load indices derived from sRPE, including training monotony and training strain, over a prolonged monitoring period of 8–12 weeks in female boxers. The majority of available research has either been conducted on male athletes or has relied on short-term observation protocols, typically spanning 1–4 weeks, thereby restricting insight into longer-term load–adaptation dynamics [18]. Moreover, when women are included in training-load research, sex-specific analyses are frequently absent or statistically underpowered, limiting the ability to derive practical, gender-responsive recommendations for training management [19]. In addition, no previous study has longitudinally monitored both wall-sit endurance and repetitive jump performance on a weekly basis. Accordingly, the simultaneous monitoring of sRPE-derived internal load indices alongside two complementary neuromuscular performance tests over a 10-week period addresses an important gap in the current literature.

Beyond documenting weekly fluctuations, the present study offers an applied contribution by proposing a practical framework for interpreting internal training load and neuromuscular performance changes within a real-world training context. By examining longitudinal patterns in sRPE-derived load indices alongside two complementary neuromuscular performance tests assessing isometric endurance and stretch–shortening cycle function, this approach extends beyond descriptive load surveillance. Rather than implying causal relationships, the findings are intended to support the contextual interpretation of within-athlete load–response patterns over time, thereby informing individualized training-load management in elite female boxers [7].

In addition to descriptive monitoring, the present design enables the examination of temporal alignment between internal load indices and neuromuscular performance across microcycles in a real-world setting. Therefore, the aim of the present study was to longitudinally monitor internal training load and neuromuscular performance over 10 weeks in elite junior female boxers, and to describe their week-to-week variations and temporal patterns within a naturalistic, observational framework.

## 2. Materials and Methods

### 2.1. Study Design

This study employed a 10-week longitudinal observational design to examine weekly fluctuations in internal training load, external neuromuscular performance, training monotony, and training strain in elite junior female boxers. No experimental manipulation was introduced. Weekly neuromuscular assessments and daily training load recordings were performed under consistent, real-world training conditions to ensure ecological validity.

### 2.2. Participants

A priori power analysis was conducted using G*Power 3.1 for a repeated-measures ANOVA with 10 measurements (f = 0.25, α = 0.05, power = 0.95, correlation among repeated measures = 0.50, ε = 1). The required sample size was N = 18; therefore, the inclusion of 20 athletes was considered sufficient. Observed effect sizes (ηp^2^ = 0.32–0.86) corresponded to post hoc power values >0.99 for all primary outcomes. Twenty elite junior female boxers (age: 18.9 ± 1.2 years; height: 165.4 ± 5.8 cm; body mass: 59.8 ± 6.1 kg; body fat: 18.7 ± 3.4%; BMI: 21.8 ± 2.1 kg/m^2^) volunteered to participate. All athletes were members of the same national high-performance boxing program and had a minimum of three years of structured training experience. Participants were required to be actively competing elite junior female boxers and to maintain regular supervised training participation (≥90% session attendance) throughout the 10-week monitoring period. Athletes had to be free from musculoskeletal injury in the previous six months. Exclusion criteria included training interruption of one week or more, failure to complete weekly neuromuscular assessments, use of medications or supplements affecting fatigue or performance, or any medical condition influencing load tolerance or physical output.

The study was approved by the Health Sciences Ethics Committee of Çankırı Karatekin University (Meeting No: 22; 9 July 2025) and conducted in accordance with the Declaration of Helsinki. All participants provided written informed consent prior to participation. Confidentiality and data privacy were maintained throughout the study, and athletes were informed of their right to withdraw at any time without consequences.

### 2.3. Training Period Structure

Although the present investigation adopted an observational design without experimental manipulation of training variables, the monitored 10-week period corresponded to a structured preparatory phase planned by the national coaching staff. This phase was situated between a general preparation stage and the final pre-competition taper, approximately 4–6 weeks prior to the target competition period, which is consistent with commonly reported preparatory timelines in elite sport periodization models [20,21]. Training organization followed commonly applied periodization principles in elite combat-sport settings, including an initial reloading phase, a progressive overload block, and a planned relative unloading microcycle, as previously described in high-performance combat-sport environments [21,22].

Importantly, interpretations of weekly “peaks,” “fatigue accumulation,” and “recovery” are grounded in this documented training context and established periodization frameworks but should not be interpreted as causal effects. Given the observational nature of the study and the absence of controlled load manipulation, these terms are used descriptively to contextualize observed week-to-week fluctuations in internal load indices and neuromuscular performance within a real-world training environment, in line with current recommendations for the interpretation of observational athlete-monitoring data [19].

### 2.4. Procedures

#### 2.4.1. Testing Environment and Standardization

All weekly assessments were conducted in the indoor facility of the national boxing center. Environmental conditions were standardized across all testing weeks, with temperature maintained at 20–22 °C and relative humidity at 40–50%. Testing occurred on the same weekday (Tuesday) and within a fixed 14:00–16:00 time window to minimize circadian influences known to affect neuromuscular performance [23]. Athletes refrained from strenuous exercise, caffeine intake, and high-intensity activities for at least 24 h prior to each assessment. All testing was supervised by the same evaluator to eliminate inter-rater variability.

##### Warm-Up Procedures

Before each testing session, athletes completed a standardized warm-up lasting approximately 13–15 min, consisting of light jogging, dynamic mobility exercises, upper-limb activation, and three submaximal practice jumps. A brief familiarization of the wall-sit position was included to reduce intra-session variability. The warm-up routine remained identical across all 10 weeks to prevent warm-up–related fluctuations in performance.

#### 2.4.2. Internal Load Monitoring

Internal load was quantified using the sRPE method. Thirty minutes after each training session, athletes reported their perceived exertion using the CR-10 scale, and daily internal load was calculated as:

sRPE load = RPE × session duration (minutes)

This method is widely validated for monitoring training stress across sports [3]. Weekly training monotony and training strain were calculated based on Foster’s model:

$$\mathrm{Monotony}\;=\frac{\mathrm{Mean}\;\mathrm{Weekly}\;\mathrm{Load}}{\mathrm{Standard}\;\mathrm{Deviation}\;\mathrm{of}\;\mathrm{Daily}\;\mathrm{Loads}}$$

Training Strain=Weekly Total Load × Monotony

#### 2.4.3. Training Exposure (Jump Counts)

Training exposure was quantified using coach-recorded jump counts collected after each conditioning or plyometric session. Weekly totals were calculated by summing all valid repetitions. Although no inertial measurement units (IMUs) were employed, coach-observed repetition counts provide ecologically valid indicators of training exposure in combat sports settings [24]. This approach is further supported by recent methodological research highlighting the use of repetition counts as contextual indicators of training exposure in applied sport monitoring environments [25]. It should be noted that jump counts were used as an indicator of training exposure rather than a measure of mechanical external load. These data reflect the volume of plyometric actions performed during training sessions and were included to provide contextual information on training content, not to quantify external load in a biomechanical or kinetic sense.

#### 2.4.4. Neuromuscular Performance Testing

##### Test Selection Rationale

The wall-sit endurance test was selected as a simple field-based assessment of lower-limb isometric endurance and peripheral fatigue tolerance, reflecting the capacity to sustain force under prolonged static loading conditions. In contrast, the repetitive jump test was included to capture neuromuscular fatigue resistance during repeated stretch–shortening cycle actions, which are highly relevant to the intermittent explosive demands of boxing. Together, these tests were chosen to provide complementary information on distinct aspects of neuromuscular function using low-cost and easily implementable assessments suitable for routine athlete monitoring [11]. This field-based neuromuscular monitoring approach is also aligned with recent recommendations supporting the use of simple performance tests for longitudinal fatigue tracking in applied sport settings [26].

##### Wall-Sit Endurance Test

Athletes performed a standardized wall-sit endurance test by maintaining a seated position with the back against a wall, knees flexed at 90°, feet shoulder-width apart, and hands resting on the thighs. Timing commenced when the correct position was achieved and terminated upon loss of posture or voluntary cessation. Performance was recorded using a digital stopwatch (CASIO HS-80TW, Japan). Wall-sit endurance measures demonstrate acceptable reliability for assessing fatigue-related changes in muscular endurance [12].

##### Repetitive Jump Test

Repetitive jump performance was assessed using a standardized continuous countermovement jump (CMJ) protocol, performed until voluntary exhaustion or until performance dropped below the predefined technical threshold. The test was executed on a flat indoor surface with athletes keeping their hands on their hips, feet shoulder-width apart and performing consistent self-selected countermovement depth.

##### Test Duration

The test was not time-limited; instead, athletes performed maximal continuous jumps until they could no longer maintain the required jump height or technical standards. This approach is consistent with fatigue-sensitive SSC protocols validated in previous neuromuscular monitoring studies [11,27].

##### Jump-Height Decline Detection

Jump height was recorded using a validated mobile application (MyJump2). A repetition was considered failed when the jump height fell ≥20% below the average height of the first three valid jumps, or when two consecutive jumps showed a progressive decline exceeding 10% each, or when technical criteria were violated (e.g., loss of hip position, arm swing, or pause >0.5 s). This threshold (≥20% drop) is widely used in repeated SSC protocols for identifying neuromuscular fatigue [27,28].

##### Invalid Repetition Criteria

A jump was classified as invalid if arm swing occurred, take-off was not fully extended, landing was excessively deep or unbalanced, or a pause of >0.5 s was observed between jumps. Correlational criteria were not used; only objective, predefined thresholds were applied. Although multiple failure criteria were applied, all thresholds were predefined, objective, and consistently enforced by the same evaluator across all testing sessions, thereby minimizing subjectivity in test termination.

##### Outcome Variable

The primary outcome was the number of valid consecutive jumps until failure. Although reliability was not tested in the present sample, previous studies have reported excellent test–retest reliability for repeated CMJ protocols (ICC = 0.85–0.95; CV = 3.5–6.5%). Therefore, while the test is considered sufficiently reliable for longitudinal monitoring in elite athletes, small week-to-week changes should be interpreted with caution.

#### 2.4.5. Training Program During Monitoring

Athletes continued their habitual training routines, typically comprising 5–6 sessions per week (90–120 min each), including technical–tactical drills, conditioning, strength training, and plyometrics, as prescribed by the national coaching staff within the ongoing preparatory phase. All completed sessions were included in load analysis; missed sessions were logged but not replaced. This approach preserved the ecological integrity of real-world training exposure during the monitoring period.

#### 2.4.6. Data Processing and Quality Control

Daily sRPE logs and jump counts were checked weekly for completeness and accuracy. Raw values were visually inspected for outliers or inconsistencies, but no smoothing, transformation, or normalization procedures were applied

### 2.5. Statistical Analysis

All statistical analyses were conducted using IBM SPSS Statistics (Version 26, IBM Corp., Armonk, NY, USA). Data distribution was assessed using the Shapiro–Wilk test and Q–Q plot inspection. Homogeneity of variances was tested using Levene’s test. Sphericity was examined using Mauchly’s test, and where violated, Greenhouse–Geisser corrections were applied.

Weekly changes in internal load (sRPE, monotony, strain) and neuromuscular performance (wall-sit time, mono-jump repetitions) were analyzed using one-way repeated-measures ANOVA with “week” (10 levels) as the within-subject factor. Significant main effects were followed by Bonferroni-adjusted pairwise comparisons. Effect sizes were reported using partial eta squared (ηp^2^) and interpreted as small (0.01), moderate (0.06), or large (0.14). Statistical significance was set at *p* < 0.05.

## 3. Results

Table 1 presents weekly changes in weighted average loss in jump performance (WALjump) across the 10-week training period in elite female boxers. WALjump was used as an integrated indicator reflecting acute and cumulative reductions in neuromuscular performance across training weeks. Results of the repeated-measures analysis of variance demonstrated a statistically significant main effect of week on WALjump values (*F*(9, 171) = 59.29, *p* < 0.001), with a large effect size (ηp^2^ = 0.757). Examination of weekly mean values showed higher WALjump values in Week 1 (337.10 ± 61.02) and Week 6 (359.77 ± 51.80), whereas the lowest WALjump value was observed in Week 7 (162.08 ± 41.51). Relatively elevated WALjump values were also observed in Week 2 (418.39 ± 48.24) and Week 4 (324.73 ± 62.12). Bonferroni-adjusted pairwise comparisons between consecutive weeks indicated statistically significant differences for all adjacent-week comparisons (all *p* < 0.001). The largest effect sizes were observed for the comparisons between Weeks 2–3 (ηp^2^ = 0.919), Weeks 5–6 (ηp^2^ = 0.877), and Weeks 6–7 (ηp^2^ = 0.924).

Table 2 presents weekly changes in weighted average session rating of perceived exertion (WAL sRPE) across the 10-week training period in elite female boxers. WAL sRPE was used as an indicator of internal training load accumulated across training sessions within each week. Results of the repeated-measures analysis of variance showed a statistically significant main effect of week on WAL sRPE values across the training period (*F*(9, 171) = 101.32, *p* < 0.001), with a large effect size (ηp^2^ = 0.842). Examination of weekly mean values indicated a progressive increase in WAL sRPE from Week 1 (1872.36 ± 231.54) to a peak in Week 6 (2856.22 ± 375.10), followed by a pronounced reduction in Week 7 (1239.48 ± 279.37). Subsequently, moderate increases were observed in Week 8 (1758.32 ± 255.01) and Week 10 (1824.91 ± 203.88). Bonferroni-adjusted pairwise comparisons between consecutive weeks revealed statistically significant differences for the majority of adjacent-week comparisons (most comparisons *p* < 0.001), with effect sizes ranging from moderate to very large across the training period.

Table 3 presents weekly changes in jump-based training monotony across the 10-week training period in elite female boxers. Monotony jumps were considered an indicator reflecting the degree of within-week variability in jump-based neuromuscular loading, with higher values indicating more uniform (monotonous) loading patterns and lower values indicating greater variability. Results of the repeated-measures analysis of variance demonstrated a statistically significant main effect of week on monotony jumps across the training period (*F*(9, 171) = 111.67, *p* < 0.001, ηp^2^ = 0.855), indicating substantial variation in monotony values across weeks. Examination of weekly mean values showed that the highest monotony value was observed in Week 2 (0.99 ± 0.11), followed by a secondary elevation in Week 7 (0.93 ± 0.15). In contrast, markedly lower monotony values were recorded in Week 5 (0.38 ± 0.00) and Week 6 (0.59 ± 0.04). The notably small standard deviation observed in Week 5 reflects limited inter-individual variability in monotony values during that week. Bonferroni-adjusted pairwise comparisons between consecutive weeks revealed statistically significant differences for the majority of adjacent-week comparisons (most comparisons *p* < 0.001), with the largest effect sizes observed between Weeks 4–5 (ηp^2^ = 0.989) and Weeks 5–6 (ηp^2^ = 0.969). In contrast, the difference between Weeks 8 and 9 was not statistically significant (*p* = 0.940).

Table 4 presents weekly changes in sRPE-derived training monotony across the 10-week training period in elite female boxers. sRPE-derived monotony was used as an indicator reflecting the degree of week-to-week uniformity in internal training load, with higher values representing more monotonous loading patterns and lower values indicating greater variability. Results of the repeated-measures analysis of variance demonstrated a statistically significant main effect of week on training monotony (*F*(9, 171) = 165.97, *p* < 0.001), with a large effect size (ηp^2^ = 0.897). Examination of weekly mean values showed higher monotony values in Week 1 (1.14 ± 0.08) and Week 2 (1.05 ± 0.08), followed by a marked reduction in Week 3 (0.65 ± 0.02) and Week 5 (0.38 ± 0.00). Subsequently, monotony values increased in Week 6 (0.80 ± 0.11) and remained at comparable levels through Weeks 7 (0.77 ± 0.13), 8 (0.70 ± 0.04), and 9 (0.79 ± 0.06), before a slight decrease in Week 10 (0.72 ± 0.02). Bonferroni-adjusted pairwise comparisons between consecutive weeks indicated statistically significant differences for the majority of adjacent-week comparisons, with the largest effect sizes observed for the comparisons between Weeks 4–5 (ηp^2^ = 0.999), Weeks 2–3 (ηp^2^ = 0.958), and Weeks 3–4 (ηp^2^ = 0.956). In contrast, differences between Weeks 6–7 (*p* = 0.379) and Weeks 7–8 (*p* = 0.060) were not statistically significant.

Table 5 presents weekly changes in jump-based training strain across the 10-week training period in elite female boxers. Jump-based strain represents a composite indicator derived from jump-based training exposure and monotony, reflecting the overall magnitude and within-week distribution of jump-based training load. Repeated-measures ANOVA demonstrated a significant main effect of week (*F*(9, 171) = 115.05, *p* < 0.001), with a very large effect size (ηp^2^ = 0.858), indicating substantial variation in jump-based strain across the monitoring period. Weekly mean strain values were highest in Week 2 (428.04 ± 77.02) and Week 1 (354.77 ± 58.10), whereas markedly lower values were observed in Week 5 (76.55 ± 15.42). During the subsequent weeks (Weeks 6–10), strain values remained at moderate levels, indicating relatively stable jump-based loading patterns. Bonferroni-adjusted pairwise comparisons revealed statistically significant differences between several consecutive weeks, particularly between Weeks 2–3, 3–4, 4–5, and 5–6 (all *p* < 0.001), with large to very large effect sizes (ηp^2^ = 0.831–0.961). In contrast, no significant differences were observed between Weeks 6–7 (*p* = 0.954) and Weeks 7–8 (*p* = 0.103), suggesting comparatively stable strain levels during these periods.

Table 6 presents weekly changes in sRPE-derived training strain across the 10-week training period in elite female boxers. sRPE-derived training strain was used as an indicator reflecting the combined magnitude and distribution of internal training load accumulated within each week. Results of the repeated-measures analysis of variance demonstrated a statistically significant main effect of week on training strain (*F*(9, 171) = 123.27, *p* < 0.001), with a large effect size (ηp^2^ = 0.866). Examination of weekly mean values showed higher training strain values in Week 2 (3734.96 ± 771.65) and Week 1 (3669.69 ± 809.37), followed by a pronounced reduction in Week 3 (1065.85 ± 69.09) and Week 5 (616.31 ± 23.99). Subsequently, training strain values increased markedly in Week 6 (2928.16 ± 593.59) and remained elevated in Weeks 7 (1166.76 ± 402.43), 8 (1357.17 ± 263.59), 9 (1656.37 ± 286.35), and 10 (1744.93 ± 142.69). Bonferroni-adjusted pairwise comparisons between consecutive weeks indicated statistically significant differences for most adjacent-week comparisons (most comparisons *p* < 0.001), with the largest effect sizes observed for the comparisons between Weeks 4–5 (ηp^2^ = 0.948), Weeks 5–6 (ηp^2^ = 0.940), and Weeks 2–3 (ηp^2^ = 0.923). In contrast, differences between Weeks 1–2 (*p* = 0.806), Weeks 7–8 (*p* = 0.108), and Weeks 9–10 (*p* = 0.252) were not statistically significant.

Table 7 presents weekly changes in acute-to-chronic workload ratio derived from jump data (ACWR jumps) across the 10-week training period in elite female boxers. ACWR jumps were used as an indicator reflecting the relationship between short-term (acute) and longer-term (chronic) jump-based neuromuscular loading. Results of the repeated-measures analysis of variance demonstrated a statistically significant main effect of week on ACWR jump values (*F*(9, 171) = 84.63, *p* < 0.001), with a large effect size (ηp^2^ = 0.817). Examination of weekly mean values showed lower ACWR jump values in Week 5 (0.65 ± 0.08) and Week 7 (0.57 ± 0.16), whereas higher values were observed in Week 6 (1.33 ± 0.12), Week 8 (1.24 ± 0.24), and Week 10 (1.26 ± 0.15). Bonferroni-adjusted pairwise comparisons between consecutive weeks indicated statistically significant differences for the majority of adjacent-week comparisons (most comparisons *p* < 0.001), with large effect sizes observed for the comparisons between Weeks 2–3 (ηp^2^ = 0.986), Weeks 5–6 (ηp^2^ = 0.971), and Weeks 6–7 (ηp^2^ = 0.930). In contrast, the difference between Weeks 3–4 was smaller (*p* = 0.045, ηp^2^ = 0.195) relative to other comparisons. These ACWR values are provided for contextual and descriptive purposes and should not be interpreted as indicators of injury risk or causal load–performance relationships.

Table 8 presents weekly changes in acute-to-chronic workload ratio derived from session rating of perceived exertion (ACWR sRPE) across the 10-week training period in elite female boxers. ACWR sRPE was used as an indicator reflecting the relationship between short-term and longer-term internal training load. Results of the repeated-measures analysis of variance demonstrated a statistically significant main effect of week on ACWR sRPE values (*F*(9, 171) = 44.83, *p* < 0.001), with a large effect size (ηp^2^ = 0.702). Examination of weekly mean values showed higher ACWR sRPE values in Weeks 1 (1.44 ± 0.05), 3 (1.49 ± 0.04), and 6 (1.35 ± 0.23), whereas lower values were observed in Weeks 4 (0.84 ± 0.04), 5 (0.75 ± 0.08), and 7 (0.69 ± 0.16). Bonferroni-adjusted pairwise comparisons between consecutive weeks indicated statistically significant differences for most adjacent-week comparisons (most comparisons *p* < 0.001), with the largest effect sizes observed for the comparisons between Weeks 3–4 (ηp^2^ = 0.994), Weeks 2–3 (ηp^2^ = 0.935), and Weeks 6–7 (ηp^2^ = 0.877). In contrast, differences between Weeks 8–9 (*p* = 0.096) and Weeks 9–10 (*p* = 0.335) were not statistically significant. These ACWR values are provided for contextual and descriptive purposes and should not be interpreted as indicators of injury risk or causal load–performance relationships.

## 4. Discussion

The present study examined weekly fluctuations in internal load and neuromuscular performance over a 10-week training period in elite junior female boxers. Internal load indicators derived from sRPE—specifically weekly monotony and strain—showed notable variations across the training weeks. Similarly, neuromuscular performance measures (wall-sit endurance and single-jump repetitions) exhibited a discernible temporal pattern, with higher values observed in week 2, followed by declines during weeks 3–5 and a partial recovery in week 6. The temporal alignment observed between training strain and jump performance may be indicative of parallel changes in perceived training stress and explosive performance capacity within this cohort, although direct within-athlete associations were not formally tested.

The week-to-week fluctuations observed in sRPE-derived load, monotony, and strain highlight the practical relevance of subjective internal load monitoring in elite female boxers. sRPE is used in athlete-monitoring research as a simple and cost-effective method for quantifying internal load [2], and associations with heart rate reserve have been reported [3]. In addition, sRPE has been shown to increase during prolonged exercise despite stable physiological intensity, indicating sensitivity to accumulated fatigue [29]. These findings support its use as a surrogate indicator of internal training stress. In the present sample, the observed fluctuations were consistent with patterns described in prolonged training environments, where accumulated load and planned recovery cycles are associated with oscillations in perceived exertion.

Training monotony and strain provided additional context regarding the weekly distribution and cumulative impact of training load. Both indices demonstrated substantial variability across the monitoring period, with weeks of higher strain temporally coinciding with lower jump performance values. Given that subjective load measures integrate psychological, emotional, and physiological dimensions of fatigue, they may be particularly sensitive to short-term changes in training demands [2,30]. Comparable temporal patterns have been reported in team and combat sports, where elevated monotony and strain have been associated with impaired well-being, altered hormonal responses, and reduced recovery capacity [6,22,31]. Overall, the observed patterns suggest that simple field-based monitoring tools can provide contextual insight when interpreted within an appropriate training framework, rather than as causal indicators. Previous research indicates that fluctuations in workload indices may co-occur with changes in neuromuscular performance, supporting the concurrent monitoring of training load and neuromuscular status within an observational framework without implying causality [32,33]. A key finding of the present study is the consistent temporal alignment observed between peaks in internal load indices, specifically training strain and monotony, and concurrent decrements in neuromuscular performance measures. ACWR values were included as contextual workload indicators rather than predictive or inferential metrics, in line with recent methodological discussions emphasizing cautious interpretation of workload ratios [34]. These patterns were evident across multiple microcycles and were not isolated to a single variable, strengthening the interpretation that the monitoring tools used were sensitive to week-to-week load dynamics in elite female boxers. However, no direct statistical analyses were conducted to examine the association between these variables. These observations should therefore be interpreted descriptively and do not imply a causal relationship. While the monitoring tools appeared sensitive to weekly fluctuations, the observational design does not permit mechanistic conclusions.

Fluctuations in wall-sit endurance and single-jump repetitions provide descriptive indication of parallel changes in training stress and neuromuscular performance across the monitoring period. Previous studies have reported that countermovement jump and other explosive-strength measures tend to decline following periods of heavy loading and may improve during reduced-load phases or tapering periods [28,32]. Research has also indicated that neuromuscular performance can be maintained or even enhanced during preparatory phases despite elevations in biochemical markers of muscle damage [33], suggesting that jump-based assessments may reflect both fatigue-related decrements and adaptive responses. Accordingly, the temporal pattern observed in the present study—characterized by declines during weeks 3–5 and partial recovery thereafter—appears consistent with these reports, although direct causal relationships cannot be inferred. Within this context, repetitive jump testing may offer a practical, field-based means of contextually tracking neuromuscular status when interpreted alongside training-load information, rather than serving as a definitive marker of fatigue or recovery.

With respect to measurement considerations, the wall-sit endurance test provides a practical and equipment-free approach for assessing lower-limb isometric fatigue tolerance. Previous research examining isometric lower-limb tasks, including wall-squat protocols, has reported moderate to excellent ICC (ranging from 0.70 to >0.90) [35,36]. Despite acceptable reliability, small week-to-week changes may fall within the typical measurement error of such field-based tests and therefore not necessarily reflect true physiological adaptations. Minor variations in wall-sit endurance should thus be interpreted with caution. The standardized protocol and consistent evaluator likely improved measurement consistency; however, the use of a single weekly trial may have reduced sensitivity to detect subtle neuromuscular changes.

The pronounced peak observed in Week 2 is consistent with training-camp structures commonly employed in elite boxing environments. During the initial phase of a preparatory block, training volume typically increases progressively while overall intensity remains submaximal, allowing athletes to re-establish neuromuscular and metabolic readiness. By the second week, many combat-sport microcycles introduce a more substantial loading stimulus. This phase is typically characterized by increased session frequency, greater technical–tactical density, and more demanding conditioning elements, as reported in longitudinal monitoring studies of combat-sport periodization [6,37]. In observational monitoring designs, early-phase performance fluctuations may occur alongside increases in internal load. However, such patterns do not necessarily indicate a direct load–performance relationship. Comparable observations have been reported in female athlete populations across different phases of the training cycle [38]. As no direct causal evidence was established, this interpretation should be understood as a context-dependent observation rather than a mechanistic conclusion.

Similar inter-individual variability is commonly observed in athlete-monitoring research, where comparable training loads may elicit different fatigue and performance responses across athletes. In female athletes, variability in subjective and objective performance across menstrual phases [13], inter-individual hormonal fluctuations [39], and inconsistent fatigue perceptions across the cycle [40] have been reported. These heterogeneous response patterns suggest that physiological and perceptual responses to training load may differ substantially between individuals, even within relatively homogeneous training groups. Additional unmonitored factors such as sleep, energy availability, and autonomic status (e.g., HRV) may also contribute to inter-individual differences in training responses. Given the multifactorial nature of sports training, which involves interactions among physical, psychological, and contextual factors, an interdisciplinary and individualized approach to athlete monitoring is often recommended to support performance development [41,42,43,44,45]. Interpretations related to fatigue, recovery, and potentiation are framed as context-dependent observations. Although consistent temporal alignment between load and performance variables was observed across the monitoring period, causal inference remains beyond the scope of the present observational design.

### 4.1. Practical Implications

The findings of this study offer several practical considerations for coaches working with elite female boxers. Training strain may serve as a feasible indicator of periods characterized by elevated perceived training stress, potentially informing decisions related to weekly workload adjustments or the emphasis on recovery strategies. Monitoring training monotony may also assist in microcycle planning by highlighting phases in which training becomes overly uniform, which could contribute to increased fatigue if sustained. In addition, incorporating simple neuromuscular assessments—such as weekly jump repetitions—may provide timely, low-cost feedback regarding changes in neuromuscular status without disrupting established training routines. Finally, the marked inter-individual variability observed in both load and performance responses underscores the importance of individualized load-management strategies, rather than uniform prescriptions, when working with elite female boxers. Collectively, these considerations support the contextual use of accessible monitoring tools to inform coaching decision-making, rather than to dictate prescriptive training adjustments.

### 4.2. Limitations and Future Directions

This study has several limitations that should be acknowledged. The sample consisted exclusively of elite junior female boxers, which limits the generalizability of the findings to other age groups, competitive levels, or male athletes. Although the 10-week monitoring period was sufficient to capture short-term fluctuations, it may not fully represent longer-term adaptation processes. Furthermore, while sRPE and neuromuscular performance tests provided meaningful descriptive indicators of internal load and functional status, no boxing-specific external load measures—such as punch frequency, contact intensity, or technical–tactical volume—were included. Future studies could integrate coach-coded actions or wearable technologies to complement subjective monitoring approaches.

In addition, several biological and contextual factors, including hormonal status, energy availability, sleep, heart rate variability, and markers of muscle damage, were not assessed. The absence of menstrual cycle monitoring represents a particularly relevant limitation in research involving female athletes, as hormonal fluctuations across follicular and luteal phases have been associated with changes in perceived exertion, fatigue, mood, thermoregulation, and, in some cases, neuromuscular performance. Although objective performance variations across menstrual phases are often small or inconsistent, the lack of cycle tracking may have contributed to some of the observed week-to-week variability, independent of training load. Future investigations should therefore consider incorporating menstrual cycle monitoring, hormonal profiling, or at minimum, self-reported cycle phase tracking to better contextualize training responses in elite female boxers.

## 5. Conclusions

This study presents findings indicating a consistent temporal alignment between internal load indices and neuromuscular performance changes over a 10-week training period in elite junior female boxers. These concurrent patterns may support the contextual evaluation of week-to-week load dynamics through simple field-based monitoring tools such as sRPE-derived metrics and jump performance tests.

Causal inferences cannot be made within this observational design. Nevertheless, the findings suggest that the combined use of subjective load measures and accessible neuromuscular assessments may be considered a practical approach for individualized athlete monitoring in female combat-sport settings.

## Figures and Tables

**Table 1:** Weekly changes in weighted average loss in jump performance (WALjump) during the 10-week training period.

	Week	Mean	S.D.	95% CI		*F*	*p.*	np^2^	Weeks	*F*1	*p*1.	np^2^-1
				**Lower Bound**	**Upper Bound**							
Weighted Average Loss in Jump Performance (WALjump)	1	337.10	61.02	308.55	365.66	59.287	<0.001	0.757	1–2	22.98	<0.001	0.547
	2	418.39	48.24	395.81	440.96				2–3	214.75	<0.001	0.919
	3	225.54	23.97	214.32	236.75				3–4	35.07	<0.001	0.649
	4	324.73	62.12	295.66	353.80				4–5	58.53	<0.001	0.755
	5	182.79	46.12	161.21	204.38				5–6	136.06	<0.001	0.877
	6	359.77	51.80	335.52	384.01				6–7	231.41	<0.001	0.924
	7	162.08	41.51	142.65	181.51				7–8	86.93	<0.001	0.821
	8	311.92	60.18	283.75	340.08				8–9	37.45	<0.001	0.663
	9	221.50	31.99	206.53	236.48				9–10	90.20	<0.001	0.826
	10	325.30	38.88	307.10	343.49							

Note. Repeated-measures ANOVA showed a significant main effect of week on weighted average loss in jump performance (WALjump), *F*(9, 171) = 59.29, *p* < 0.001, ηp^2^ = 0.757. Bonferroni-adjusted pairwise comparisons between consecutive weeks are reported in this table.

**Table 2:** Weekly changes in weighted average sRPE (WAL sRPE) during the 10-week training period.

	Week	Mean	S.D.	95% CI		*F*	*p*.	np^2^	Weeks	*F*1	*p*1.	np^2^-1
				**Lower Bound**	**Upper Bound**							
Wal sRPE	1	3322.36	487.43	3094.24	3550.48	101.317	<0.001	0.842	1–2	3.80	0.066	0.167
	2	3023.80	452.92	2811.83	3235.78				2–3	186.77	<0.001	0.908
	3	1575.05	156.04	1502.02	1648.08				3–4	46.22	<0.001	0.709
	4	1933.12	230.97	1825.02	2041.21				4–5	38.49	<0.001	0.669
	5	1611.31	159.13	1583.64	1638.98				5–6	184.22	<0.001	0.907
	6	2856.22	375.10	2680.67	3031.77				6–7	276.29	<0.001	0.936
	7	1239.48	279.37	1108.74	1370.23				7–8	33.03	<0.001	0.635
	8	1758.32	255.01	1638.97	1877.67				8–9	16.44	0.001	0.464
	9	2130.10	300.96	1989.25	2270.95				9–10	3.83	0.065	0.168
	10	2261.40	176.61	2178.74	2344.06							

Note. Repeated-measures ANOVA showed a significant main effect of week on weighted average sRPE (WAL sRPE), *F*(9, 171) = 101.32, *p* < 0.001, ηp^2^ = 0.842. Bonferroni-adjusted pairwise comparisons between consecutive weeks are reported in this table.

**Table 3:** Weekly changes in jump-based training monotony during the 10-week training period.

	Week	Mean	S.D.	95% CI		*F*	*p*.	np^2^	Weeks	*F*1	*p*1	np^2^-1
				**Lower Bound**	**Upper Bound**							
Monotony jumps	1	0.84	0.08	0.80	0.87	111.673	<0.001	0.855	1–2	55.20	<0.001	0.744
	2	0.99	0.11	0.93	1.04				2–3	166.38	<0.001	0.898
	3	0.66	0.02	0.65	0.67				3–4	16.16	0.001	0.460
	4	0.70	0.03	0.68	0.71				4–5	1662.07	<0.001	0.989
	5	0.38	0.00	0.38	0.38				5–6	598.63	<0.001	0.969
	6	0.59	0.04	0.57	0.61				6–7	76.575	<0.001	0.801
	7	0.93	0.15	0.86	1.00				7–8	35.23	<0.001	0.650
	8	0.72	0.05	0.70	0.74				8–9	0.006	0.940	<0.001
	9	0.72	0.05	0.69	0.74				9–10	43.71	<0.001	0.697
	10	0.64	0.04	0.62	0.65							

Note: Repeated-measures ANOVA revealed a significant main effect of week (*F*(9, 171) = 111.67, *p* < 0.001, ηp^2^ = 0.855). Bonferroni-adjusted pairwise comparisons between consecutive weeks are reported in this table.

**Table 4:** Weekly changes in sRPE-derived training monotony during the 10-week training period.

	Week	Mean	S.D.	95% CI		*F*	*p*.	np^2^	Weeks	*F*1	*p*1.	np^2^-1
				**Lower Bound**	**Upper Bound**							
Monotony jumps	1	1.14	0.08	1.10	1.18	165.967	<0.001	0.897	1–2	12.48	0.002	0.396
	2	1.05	0.08	1.01	1.08				2–3	430.28	<0.001	0.958
	3	0.65	0.02	0.64	0.66				3–4	415.06	<0.001	0.956
	4	0.75	0.01	0.74	0.76				4–5	14674.71	<0.001	0.999
	5	0.38	0.00	0.38	0.38				5–6	275.95	<0.001	0.936
	6	0.80	0.11	0.75	0.86				6–7	0.81	0.379	0.041
	7	0.77	0.13	0.71	0.83				7–8	4.02	0.060	0.174
	8	0.70	0.04	0.68	0.72				8–9	30.56	<0.001	0.617
	9	0.79	0.06	0.76	0.82				9–10	22.83	<0.001	0.546
	10	0.72	0.02	0.71	0.73							

**Note.** Repeated-measures analysis of variance showed a statistically significant main effect of week on sRPE-derived training monotony, *F*(9, 171) = 165.97, *p* < 0.001, with a large effect size (ηp^2^ = 0.897). Bonferroni-adjusted pairwise comparisons between consecutive weeks are reported in the table.

**Table 5:** Weekly changes in jump-based training strain during the 10-week training period (n = 20).

	Week	Mean	S.D.	95% CI		*F*	*p*.	np^2^	Weeks	*F*1	*p*1.	np^2^-1
				Lower Bound	Upper Bound							
Monotony jumps	1	354.77	58.10	327.57	381.96	115.05	<0.001	0.858	1–2	9.60	0.006	0.336
	2	428.04	77.02	391.99	464.08				2–3	233.94	<0.001	0.925
	3	145.97	15.56	138.69	153.26				3–4	93.17	<0.001	0.831
	4	228.78	33.04	213.32	244.24				4–5	463.13	<0.001	0.961
	5	76.55	15.42	69.33	83.76				5–6	192.15	<0.001	0.910
	6	211.63	33.48	195.96	227.30				6–7	0.003	0.954	0.000
	7	210.99	46.50	189.23	232.75				7–8	2.937	0.103	0.134
	8	232.49	38.09	214.66	250.32				8–9	21.66	<0.001	0.533
	9	172.04	28.74	158.59	185.49				9–10	25.363	<0.001	0.572
	10	216.38	22.51	205.85	226.92							

Note. Repeated-measures analysis of variance showed a statistically significant main effect of week on jump-based training strain, *F*(9, 171) = 115.05, *p* < 0.001, with a large effect size (ηp^2^ = 0.858). Bonferroni-adjusted pairwise comparisons between consecutive weeks are reported in the table.

**Table 6:** Weekly changes in sRPE-derived training strain during the 10-week training period.

	Week	Mean	S.D.	95% CI		*F*	*p*.	np^2^	Weeks	*F*1	*p*1.	np^2^-1
				**Lower Bound**	**Upper Bound**							
Monotony jumps	1	3669.69	809.37	3290.89	4048.49	123.271	<0.001	0.866	1–2	0.062	0.806	0.003
	2	3734.96	771.65	3373.82	4096.11				2–3	226.72	<0.001	0.923
	3	1065.85	69.09	1033.52	1098.18				3–4	85.41	<0.001	0.818
	4	1425.91	190.41	1336.80	1515.03				4–5	348.49	<0.001	0.948
	5	616.31	23.99	605.08	627.53				5–6	297.73	<0.001	0.940
	6	2928.16	593.59	2650.35	3205.97				6–7	85.45	<0.001	0.818
	7	1166.76	402.43	978.42	1355.11				7–8	2.84	0.108	0.130
	8	1357.17	263.59	1233.81	1480.54				8–9	14.77	0.001	0.437
	9	1656.37	286.35	1522.36	1790.39				9–10	1.39	0.252	0.068
	10	1744.93	142.69	1678.15	1811.71							

Note. Repeated-measures analysis of variance showed a statistically significant main effect of week on sRPE-derived training strain, *F*(9, 171) = 123.27, *p* < 0.001, with a large effect size (ηp^2^ = 0.866). Bonferroni-adjusted pairwise comparisons between consecutive weeks are reported in the table.

**Table 7:** Weekly changes in ACWR jumps during the 10-week training period.

	Week	Mean	S.D.	95% CI		*F*	*p*.	np^2^	Weeks	*F*1	*p*1.	np^2^-1
				**Lower Bound**	**Upper Bound**							
Monotony jumps	1	0.89	0.03	0.88	0.91	84.631	<0.001	0.817	1–2	27.68	<0.001	0.593
	2	0.87	0.02	0.85	0.88				2–3	1375.83	<0.001	0.986
	3	0.98	0.02	0.97	0.99				3–4	4.61	0.045	0.195
	4	0.94	0.08	0.90	0.98				4–5	120.15	<0.001	0.863
	5	0.65	0.08	0.61	0.69				5–6	629.28	<0.001	0.971
	6	1.33	0.12	1.28	1.39				6–7	252.84	<0.001	0.930
	7	0.57	0.16	0.50	0.65				7–8	86.43	<0.001	0.820
	8	1.24	0.24	1.13	1.35				8–9	24.91	<0.001	0.567
	9	0.92	0.09	0.88	0.96				9–10	52.87	<0.001	0.736
	10	1.26	0.15	1.18	1.33							

Note. Repeated-measures analysis of variance showed a statistically significant main effect of week on ACWR jumps, *F*(9, 171) = 84.63, *p* < 0.001, with a large effect size (ηp^2^ = 0.817). Bonferroni-adjusted pairwise comparisons between consecutive weeks are reported in the table.

**Table 8:** Weekly changes in ACWR sRPE during the 10-week training period.

	Week	Mean	S.D.	95% CI		*F*	*p*.	np^2^	Weeks	*F*1	*p*1.	np^2^-1
				**Lower Bound**	**Upper Bound**							
Monotony jumps	1	1.44	0.05	1.41	1.46	44.832	<0.001	0.702	1–2	38.54	<0.001	0.670
	2	1.39	0.03	1.38	1.41				2–3	275.42	<0.001	0.935
	3	1.49	0.04	1.47	1.51				3–4	3085.04	<0.001	0.994
	4	0.84	0.04	0.83	0.86				4–5	22.40	<0.001	0.541
	5	0.75	0.08	0.71	0.78				5–6	133.06	<0.001	0.875
	6	1.35	0.23	1.25	1.46				6–7	135.10	<0.001	0.877
	7	0.69	0.16	0.61	0.76				7–8	21.71	<0.001	0.533
	8	0.93	0.18	0.85	1.02				8–9	3.06	0.096	0.139
	9	1.14	0.42	0.94	1.34				9–10	0.98	0.335	0.049
	10	1.27	0.32	1.11	1.42							

Note. Repeated-measures analysis of variance showed a statistically significant main effect of week on ACWR sRPE, *F*(9, 171) = 44.83, *p* < 0.001, with a large effect size (ηp^2^ = 0.702). Bonferroni-adjusted pairwise comparisons between consecutive weeks are reported in the table.

## Data Availability

The data presented in this study are available from the corresponding author upon reasonable request.

## References

[1] Mountjoy M., Ackerman K.E., Bailey D.M., Burke L.M., Constantini N., Hackney A.C., Heikura I.A., Melin A., Pensgaard A.M., Stellingwerff T. (2023). 2023 International Olympic Committee’s (IOC) Consensus Statement on Relative Energy Deficiency in Sport (REDs). Br. J. Sports Med..

[2] Saw A.E., Main L.C., Gastin P.B. (2016). Monitoring the Athlete Training Response: Subjective Self-Reported Measures Trump Commonly Used Objective Measures: A Systematic Review. Br. J. Sports Med..

[3] Foster C., Rodriguez-Marroyo J.A., de Koning J.J. (2017). Monitoring Training Loads: The Past, the Present, and the Future. Int. J. Sports Physiol. Perform..

[4] Clemente F.M., Clark C., Castillo D., Sarmento H., Nikolaidis P.T., Rosemann T., Knechtle B. (2019). Variations of training load, monotony, and strain and dose-response relationships with maximal aerobic speed, maximal oxygen uptake, and isokinetic strength in professional soccer players. PLoS ONE.

[5] Andersen T.R., Kästner B., Arvig M., Larsen C.H., Madsen E.E. (2023). Monitoring load, wellness, and psychological variables in female and male youth national team football players during international and domestic playing periods. Front. Sports Act. Living.

[6] Nobari H., Oliveira R., Clemente F.M., Adsuar J.C., Pérez-Gómez J., Carlos-Vivas J., Brito J.P. (2020). Relationships between Training Load and Wellness Measures in Young Male Soccer Players during a Season. Int. J. Environ. Res. Public Health.

[7] Impellizzeri F.M., Tenan M.S., Kempton T., Novak A., Coutts A.J. (2020). Acute:Chronic Workload Ratio: Conceptual Issues and Fundamental Pitfalls. Int. J. Sports Physiol. Perform..

[8] Dalen-Lorentsen T., Andersen T.E., Bjørneboe J.A., Vagle M., Martin K.N., Kleppen M., Fagerland M.W., Clarsen B. (2021). A Cherry, Ripe for Picking: The Relationship Between the Acute-Chronic Workload Ratio and Health Problems. J. Orthop. Sports Phys. Ther..

[9] Dinu D., Louis J. (2020). Biomechanical Analysis of the Cross, Hook, and Uppercut in Junior vs. Elite Boxers: Implications for Training and Talent Identification. Front. Sports Act. Living.

[10] López-Laval I., Cirer-Sastre R., De Pariza J.L.A.L., Corbi F., Calleja-González J., Sitko S. (2022). Concurrent validity and reliability of an accelerometer to assess punching velocity in boxers. Gazz. Medica Ital. Arch. Sci. Med..

[11] Claudino J.G., Cronin J., Mezêncio B., McMaster D.T., McGuigan M., Tricoli V., Amadio A.C., Serrão J.C. (2017). The Countermovement Jumps to Monitor Neuromuscular Status: A Meta-Analysis. J. Sci. Med. Sport.

[12] Lea J.W.D., O’Driscoll J.M., Coleman D.A., Wiles J.D. (2021). Validity and Reliability of RPE as a Measure of Intensity during Isometric Wall Squat Exercise. J. Clin. Transl. Res..

[13] Carmichael M.A., Thomson R.L., Moran L.J., Wycherley T.P. (2021). The Impact of Menstrual Cycle Phase on Athletes’ Performance: A Narrative Review. Int. J. Environ. Res. Public Health.

[14] Paludo A.C., Paravlic A., Dvořáková K., Gimunová M. (2022). The effect of menstrual cycle on perceptual responses in athletes: A systematic review with meta-analysis. Front. Psychol..

[15] Rico-González M., Oliveira R., González Fernández F.T., Clemente F.M. (2023). Acute:Chronic Workload Ratio and Training Monotony Variations over the Season in Youth Soccer Players: A Systematic Review. Int. J. Sports Sci. Coach..

[16] Cui W., Chen Y., Wang D. (2024). The effect of optimal load training on punching ability in elite female boxers. Front. Physiol..

[17] Niu Z., Huang Z., Zhao G., Chen C. (2024). Impact of three weeks of integrative neuromuscular training on the athletic performance of elite female boxers. PeerJ.

[18] Slimani M., Miarka B., Briki W., Cheour F. (2017). Comparison of Perceived Training Load, Session Rating of Perceived Exertion, and Heart-Rate-Based Methods in Monitoring Training Load in Combat Sports. J. Strength Cond. Res..

[19] McCabe D., Martin D., McMahon G. (2023). Training load is correlated with changes in creatine kinase and wellness over a 12-week multi-stage preparatory training block for a major competition in international boxers. Physiologia.

[20] Turner A. (2011). The Science and Practice of Periodization: A Brief Review. Strength Cond. J..

[21] Issurin V.B. (2016). Benefits and Limitations of Block Periodized Training Approaches to Athletes’ Preparation: A Review. Sports Med..

[22] Kirk C., Hurst H.T., Atkins S., Kinchington M., Piggott D. (2021). The Effect of Training Load on Mixed Martial Arts Performance: An Exploratory Study. Int. J. Sports Physiol. Perform..

[23] Chtourou H., Souissi N. (2012). The Effect of Training at a Specific Time of Day. J. Strength Cond. Res..

[24] Chaabène H., Negra Y., Bouguezzi R., Capranica L., Franchini E., Prieske O., Hbacha H., Granacher U. (2018). Tests for the Assessment of Sport-Specific Performance in Olympic Combat Sports: A Systematic Review with Practical Recommendations. Front. Physiol..

[25] Bouafif N., van den Tillaar R., Mahmoudi A., Chaouachi A., Hammami R. (2026). Effects of Plyometric Jump Training on Measures of Physical Fitness and Lower-Limb Asymmetries in Male Soccer Players According to Maturity: A Randomized Controlled Trial. PLoS ONE.

[26] Geantă V.A., de Hillerin P.J., Iacobini A.R., Camenidis C.M., Ionescu A. (2025). Differences in Average Power Output Values from Computational Models of Repeated Vertical Jump Tests: A Single-Group Quasi Experimental Approach. J. Funct. Morphol. Kinesiol..

[27] Thomas K., Brownstein C.G., Dent J., Parker P., Goodall S., Howatson G., Hunter A.M. (2018). Neuromuscular Fatigue and Recovery After Heavy Resistance, Sprint, and Plyometric Exercise. Int. J. Sports Physiol. Perform..

[28] Philipp N., Held S., Behringer M., Donath L. (2024). Neuromuscular Performance Responses to Training Load in Elite Female Basketball Players: Countermovement Jump and 10–5 Repeated Jump Test Sensitivity. Int. J. Sports Physiol. Perform..

[29] Fusco A., Knutson C., King C., Mikat R.P., Porcari J.P., Cortis C., Foster C. (2020). Session RPE During Prolonged Exercise Training. Int. J. Sports Physiol. Perform..

[30] Coyne J.O.C., Coutts A.J., Newton R.U., Haff G.G., Hopkins W.G. (2018). The Current State of Subjective Training Load Monitoring—A Practical Perspective and Call to Action. Sports Med. Open.

[31] Campos F.A.D., Bertuzzi R., Dourado A.C., Santos V.G.F., Franchini E. (2020). Energy Demands and Training Load in Brazilian Jiu-Jitsu Athletes. J. Strength Cond. Res..

[32] Merrigan J.J., Cormack S.J., Chapman D.W., Newton R.U. (2021). Progression in Jump Performance Measures During Intensified Training in Team Sport Athletes. J. Sci. Med. Sport.

[33] Berriel G.P., Costa B.D.V., dos Santos J.W., Pochmann D., Nunes G.N. (2020). Neuromuscular Performance and Biochemical Responses during Preseason Training in Elite Volleyball Players. J. Strength Cond. Res..

[34] Pinelli S., Mandorino M., Fantozzi S., Lacome M. (2025). Exploring the Relationship Between the Acute:Chronic Workload Ratio and Running Parameters in Elite Football Athletes. Appl. Sci..

[35] Souron R., Colard J., Ruiz-Cárdenas J.D., Beltran A., Duché P., Gruet M. (2024). Development and Assessment of Test–Retest Reliability of a New Field Test to Evaluate Lower-Limb Muscle Fatigability in Young Adults. Mov. Sport Sci.—Sci. Motricité.

[36] Franceschi A., Robinson M.A., Owens D., Brownlee T., Ferrari Bravo D., Enright K. (2023). Reliability and Sensitivity to Change of Post-Match Physical Performance Measures in Elite Youth Soccer Players. Front. Sports Act. Living.

[37] Yildiz M., Akyildiz Z., Gunay M., Clemente F.M. (2024). Relationship Between Training Load, Neuromuscular Fatigue, and Daily Well-Being in Elite Young Wrestlers. Res. Q. Exerc. Sport.

[38] Ishida A., Bazyler C.D., Sayers A.L., Stone M.H., Gentles J.A. (2021). Seasonal Changes and Relationships in Training Loads, Neuromuscular Performance, and Recovery and Stress State in Competitive Female Soccer Players. Front. Sports Act. Living.

[39] Dudek S., Koziak W., Kornacka A., Bętkowska A., Makieła M., Dudek W., Szostak K., Tomaka R., Byra A. (2025). The Impact of the Menstrual Cycle on Sports Performance: A Narrative Review. Qual. Sport.

[40] Dhahbi W., Materne O., Chamari K. (2025). Rethinking knee injury prevention strategies: joint-by-joint training approach paradigm versus traditional focused knee strengthening. Biol. Sport.

[41] Kryeziu A.R., Iseni A., Teodor D.F., Croitoru H., Badau D. (2023). Effect of 12 Weeks of the Plyometric Training Program Model on Speed and Explosive Strength Abilities in Adolescents. Appl. Sci..

[42] Gurses V.V., Ceylan B., Sakir M., Baydil B., Hussein H.A., Badau D. (2018). Dehydration and Acute Weight Gain of Athletes Before Sport Competitions. Rev. Chim..

[43] Badau D., Steff N., Badau A., Stoica M. (2025). Enhancing Lower-Limb Simple and Choice Reaction Time and Spatial Orientation in Junior Basketball Players by Implementing Fitlight Technology in Sports Training. Percept. Mot. Ski..

[44] Stefanica V., Ceylan H.İ., Mihailescu L., Badau D., Joksimović M., Rosu D. (2025). Impact of CrossFit Intervention on Mental Health and Well-Being Among First-Year Law Students. Humanit. Soc. Sci. Commun..

[45] Moraru L., Mirea O., Toader D., Berceanu M., Soldea S., Munteanu A., Donoiu I., Raicea V. (2024). Lower Limit of Normality of Segmental Multilayer Longitudinal Strain in Healthy Adult Subjects. J. Cardiovasc. Dev. Dis..

